# Hepatic platelet and leukocyte adherence during endotoxemia

**DOI:** 10.1186/cc3968

**Published:** 2006-01-09

**Authors:** Roland S Croner, Elfie Hoerer, Yakup Kulu, Tilo Hackert, Martha-Maria Gebhard, Christian Herfarth, Ernst Klar

**Affiliations:** 1 Department of Surgery, University of Erlangen-Nuernberg, Germany; 2 Department of Surgery, University of Heidelberg, Germany; 3 Department of Experimental Surgery, University of Heidelberg, Germany; 4 Department of Surgery, University of Rostock, Germany

## Abstract

**Introduction:**

Liver microcirculation disturbances are a cause of hepatic failure in sepsis. Increased leukocyte-endothelial interaction, platelet adherence and impaired microperfusion cause hepatocellular damage. The time course and reciprocal influences of ongoing microcirculatory events during endotoxemia have not been clarified.

**Methods:**

Male Wistar rats (232 ± 17 g) underwent cecal ligation and puncture (CLP). Intravital microscopy (IVM) was performed 0, 1, 3, 5, 10 and 20 hours after CLP. Mean erythrocyte velocity, leukocyte and platelet rolling in postsinusoidal venules and sticking of leukocytes and platelets in postsinusoidal venules and hepatic sinusoids were determined. Heart rate (HR), mean arterial pressure (MAP) and portal venous blood flow (PBF) were measured. Blood count and investigation of hepatic enzyme release was performed after each IVM time point.

**Results:**

Hepatic platelet-endothelial adherence in liver sinusoids and postsinusoidal venules occurred one hour after the induction of endotoxemia. Leukocyte-endothelial interaction started three to five hours after CLP. A decrease of hepatic microperfusion could be observed at three hours in sinusoids and ten hours in postsinusoidal venules after CLP, although PBF was reduced one hour after CLP. HR remained stable and MAP decreased ten hours after CLP. Hepatic enzymes in blood were significantly elevated ten hours after CLP.

**Conclusion:**

Hepatic platelet-endothelial interaction is an early event during endotoxemia. Leukocyte adherence occurs later, which underlines the probable involvement of platelets in leukocyte recruitment. Although PBF is reduced immediately after CLP, the later onset of hepatic microperfusion decrease makes the existence of autoregulatory liver mechanisms likely.

## Introduction

The liver has a central regulatory role in metabolism and host defense mechanisms during the course of sepsis [1]. Nevertheless, hepatocellular dysfunction occurs in early stages of the disease. The release of cytokines such as tumour necrosis factor-alpha from activated Kupffer cells is one cause of cytotoxic effects on hepatocytes [1-3]. But the release and expression of endothelial adhesion molecules is also initiated by proinflammatory cytokines [1,2,4-6].

E- and P-selectins, which are expressed by activated endothelial cells, lead to the transient and reversible adhesion of leukocytes (rolling) to the endothelial surface via L-selectin [7-9]. The adhesion of platelets to endothelial cells is also mediated by selectins [10]. Activated endothelial cells produce chemoattractants, such as interleukin-8 and platelet-activating factor, that may be secreted or remain surface bound. In leukocytes, interleukin-8, platelet-activating factor and C5a initiate a cascade of intracellular events that lead to the activation of β-integrins (LFA-1 and Mac-1) [11,12]. These β-integrins enable leukocytes to adhere to endothelial adhesion molecules, such as intercellular adhesion molecules, vascular cell adhesion molecule-1 and platelet-endothelial cell adhesion molecule-1, which initiates extravasation [9,13,14]. The release of superoxide, arachidonic acid metabolites and proteases of transendothelial migrated leukocytes and the impaired microperfusion injures hepatocytes [13,15-20].

The time course of ongoing hepatic microcirculatory events during sepsis, especially the role of platelets, is not yet completely clarified. For this reason, we investigated the time dependent events of leukocyte adherence, platelet adherence and impaired microperfusion in an animal model of sepsis by intravital microscopy (IVM).

## Materials and methods

### Animals and protocols

All experimental procedures and protocols used in this investigation were approved by the Governmental Animal Protection Committee (Karlsruhe, Germany).

Male Wistar rats (232 ± 17 g) were anaesthetized by intraperitoneal injection of 20 mg/kg body weight sodium pentobarbital (Nembutal; Sanofi, Düsseldorf, Germany) and 30 mg/kg body weight intramuscular injection of Ketamin. The right jugular vein was cannulated for the infusion of reagents. Sepsis was induced by cecal ligation and puncture (CLP) [21,22]. Laparotomy of 2 cm in the lower abdomen was performed and the cecum was exteriorised. After non-obstructive ligation of the cecum, two stitches with an 18G needle were performed. The right carotid artery was cannulated for the measurement of heart rate and mean arterial pressure (MAP). To maintain anaesthesia during the observation period, the left femoral vein was cannulated for continuous sodium pentobarbital (8 mg/h/kg body weight) and Ketamin (4 mg/h/kg body weight) infusion. Rectal temperature was measured and maintained at 37°C using a heating pad.

IVM was performed in eight animals of each group immediately (0 h) and 1 h, 3 h, 5 h, 10 h and 20 h after CLP. After the IVM blood count in venous blood was performed, hepatocellular enzyme release (AST, ALT), albumin and bilirubin levels in blood, heart rate and MAP were measured. The blood flow of the portal vein (PBF) was determined using the flow probe of a small animal ultrasonic flowmeter (Transonic Systems, New York, USA [16].

### Intravital microscopy

After placing the animal beneath the microscope, a 30 minute stabilisation period followed. The upper surface of the left liver lobe was exteriorised on a specially designed mechanical stage. To maintain body temperature, the liver lobe was continuously superfused by thermostat-controlled (37.0°C) Ringer solution. Hepatic microcirculation was oserved using a specially designed microscope for epi-illumination (Orthoplan; Leica, Wetzlar, Germany; lens with 40-fold magnification, Archoplan 40/0.75 W; Zeiss, Jena, Germany). To protect the liver lobe from heat, a heat protection filter (KG 1; Leica) was located in the body of the microscope. Microscopic images were transferred to a monitor (PVM 1444QM; Sony Corp., Tokyo, Japan) by a low light camera (Kappa CF 8/1; Kappa Messtechnik, Gleichen, Germany) and recorded on a video tape for later evaluation using a computer assisted system for microcirculation analysis (Cap image; Zeintl, Heidelberg, Germany).

### Platelet preparation

Whole heparine-blood (1 ml) from donor rats was collected and platelets were stained with rhodamine 6G (Sigma Chemical, St. Louis, USA) as described elsewhere [23]. The collected blood was diluted with Alserver's buffer after addition of prostaglandin E_1_. Following a four-cycle washing procedure in phosphate-buffered saline, platelets were separated and injected in septic animals prior to IVM.

### Analysis of leukocyte-endothelial and platelet-endothelial interactions

Leukocytes were visualized by staining them with rhodamin 6G (0.1 μg/kg body weight). The leukocyte-endothelial and platelet-endothelial interactions were investigated in separate animal groups and analysed in each animal within a minimum of 10 hepatic lobuli and 10 postsinusoidal venules. Adherent leukocytes and platelets that did move or detach from the endothelium prior to a period of 30 s were defined as 'rollers'. Those that adhered to the endothelial wall for longer were classified as 'stickers'. The number of rollers and stickers in postsinusoidal venules were calculated per mm^2 ^of endothelial surface (length of observed vessel segment × diameter × π = rollers or stickers per mm^2^). Sticking leukocytes and platelets in sinusoids were quantified as stickers per mm^2 ^liver surface. Thrombotic sinusoids were calculated as not perfused sinusoids/sinusoids in hepatic lobuli (%).

### Analysis of blood flow in liver sinusoids and postsinusoidal venules

Erythrocytes from seperate donor rats were labelled with fluorescein isothiocyanate (FITC, Isomer I, No. F-7250; Sigma Chemical, Deisenhofen, Germany). Blood was washed three times with Alserver's buffer solution and one time with bicine-saline buffer solution to remove plasma. The washed erythrocytes were diluted 1:2 with bicine-saline buffer solution and incubated with FITC (9 mg/ml erythrocytes) for 180 minutes at 25°C. Labelled erythrocytes were further washed five times in bicine-saline buffer solution. Then the erythrocytes were diluted with saline until the hematocrit was 50% in citrate-phosphate-dextrose solution (No.C-7165, Sigma Chemical, Germany). Thirty minutes prior to IVM, the animals received 1.0 ml/kg bodyweight FITC-labelled erythrocytes. For the measurement of sinusoidal perfusion, the velocity of 50 erythrocytes in 10 acini was measured and calculated as a mean of erythrocyte velocyte per mm^2 ^liver surface. The velocity of 10 erythrocytes in 10 postsinusoidal venules was measured and calculated as a mean of erythrocyte velocity (MEV) per mm^2 ^of endothelial surface.

### Statistical analysis

All data are presented as mean ± standard error of the mean (SEM). Differences were considered significant for *p* < 0.05. Comparisons between groups were performed by one-way ANOVA followed by LSD test after Shapiro-Wilk's analysis for normal distribution.

## Results

### Macrohemodynamic parameters and laboratory values

The heart rate remained stable during the whole IVM investigation period. The MAP decreased significantly 10 h after CLP while the PBF was decreased significantly at 1 h after CLP compared to at 0 h (Table 1). Animals had developed significant leukopenia 3 h, 5 h, 10 h and 20 h after CLP compared to at 0 h. Platelets decreased significantly 3 h, 10 h and 20 h versus 0 h after CLP. Hematokrit remained stable during the whole investigation period. Significantly, hepatozellular enzyme liberation (AST, ALT) was detected at 10 h and 20 h after CLP compared to at 0 h. Bilirubin in blood was increased significantly at 20 h versus 0 h after CLP. Levels of albumin in blood were reduced at 3 h, 10 h and 20 h versus 0 h after CLP (Table 2).

### Intravital microscopy

**Figure 1:**
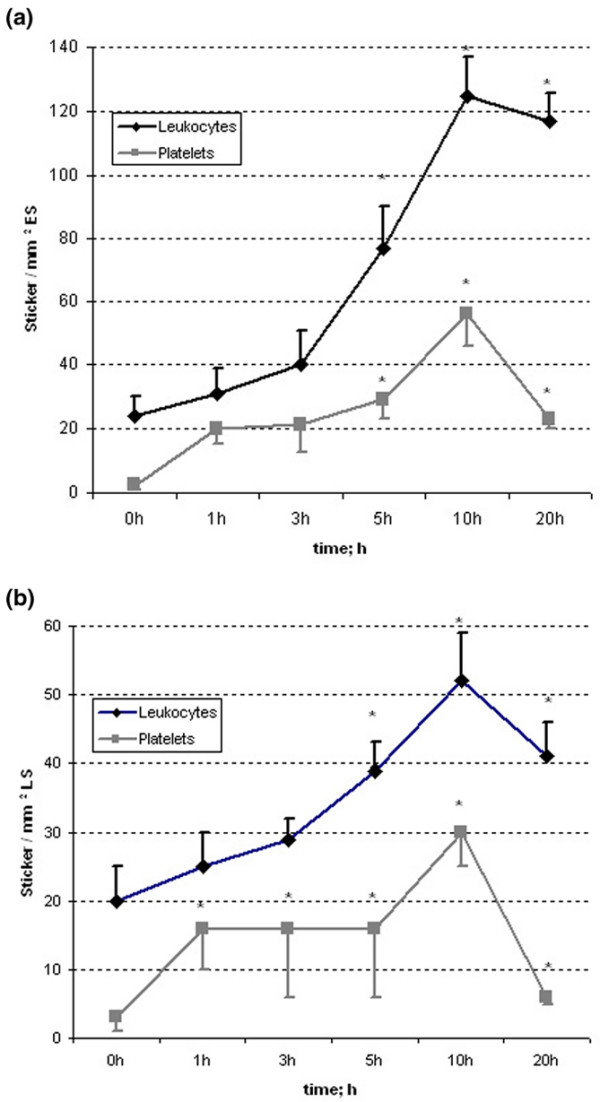
Sticking leukocytes and platelets in **(a) **postsinusoidal venules and **(b) **hepatic sinusoids. Asterisks indicate *p* < 0.05 versus 0 h. ES, endothelial surface; LS, liver surface.

**Figure 2:**
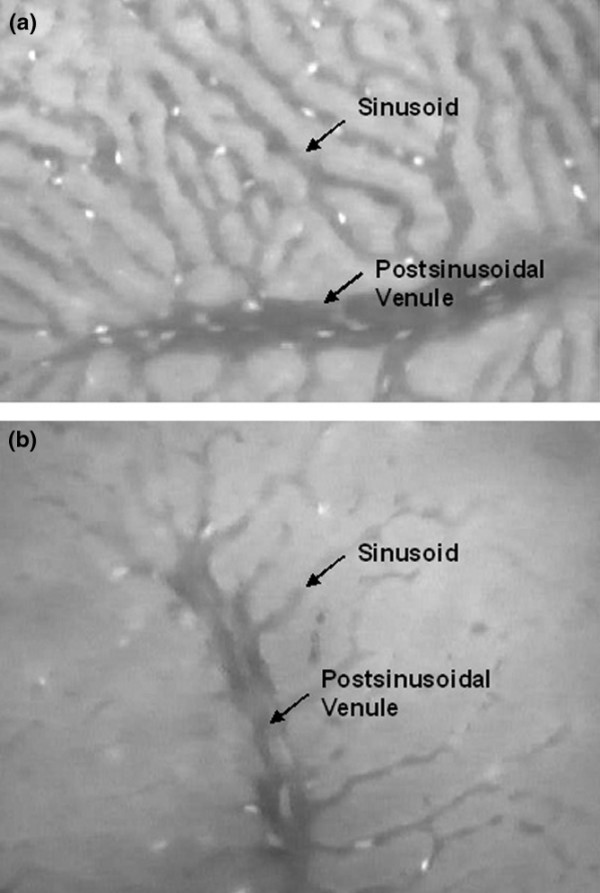
Hepatic sinusoids and postsinusoidal venules during intravital microscopy **(a) **0 h and **(b) **20 h after CLP demonstrate the decrease of perfused sinusoids and sinusoidal diameter during endotoxemia.

The MEV in hepatic sinusoids was decreased significantly at 3 h, 5 h, 10 h and 20 h versus 0 h of IVM measurement. MEV in postsinusoidal venules was decreased significantly 10 h and 20 h versus 0 h after CLP. Leukocyte rolling in postsinusoidal venules was significantly increased 3 h, 10 h and 20 h versus 0 h while platelet rolling was significantly increased at 1 h, 3 h, 5 h, 10 h, and 20 h versus 0 h of IVM. At 5 h, 10 h and 20 h versus 0 h, significantly increased sticking of leukocytes and platelets occurred in postsinusoidal venules. In hepatic sinusoids at 1 h, 3 h, 5 h, 10 h and 20 h, significantly elevated amounts of sticking platelets were detected compared to at 0 h. Sticking leukocytes in liver sinusoids were significantly increased 5 h, 10 h and 20 h versus 0 h after CLP. The sinusoidal diameter was significantly reduced 10 h and 20 h versus 0 h of IVM and the ratio of non-perfused thrombotic sinusoids was significantly increased 5 h, 10 h and 20 h versus 0 h after CLP (Table 3, Figure 1). Figure 2 shows IVM pictures of hepatic postsinusoidal venules and sinusoids and gives a visual idea of ongoing microcirculatory disturbances. The decrease of sinusoidal diameter and reduced amount of perfused sinusoids at 20 h (Figure 2b) versus 0 h (Figure 2a) are obvious.

## Discussion

### Animal model

It has been demonstrated in the CLP sepsis model that the hyperdynamic state of sepsis persists from 2 to 10 h and the hypodynamic state occurs 16 to 20 h after CLP, depending on the lesion in the cecum [22]. In our animal model, heart rate was stable during the whole investigation period while MAP decreased 10 h after CLP, which is an indicator for the occurrence of hypodynamic sepsis. Leukopenia and reduced levels of platelets in blood, which were detected 3 h after CLP, reflect signs of sepsis as they could be detected under clinical conditions. Albumin levels decreased 3 h after CLP and hepatocellular enzymes in blood increased significantly 10 h after CLP, reflecting the hepatocyte damage as one characteristic of the multiple organ dysfunction syndrome, similar to clinical findings [24,25].

### Liver perfusion

Heart rate remained stable during the IVM investigation, while MAP decreased significantly 10 h after CLP. Hypotension, which is observed in late, hypodynamic stages of sepsis, may reflect progression of the ongoing disease [24]. The PBF was significantly reduced 1 h after CLP. One reason for this finding could be the release of nitric oxide from intestinal inflammatory cells, which causes vasodilatation of intestinal blood vessels [17]. The increase of vessel diameter without adequate adaptation of cardiac output could be the reason for diminished PBF [15,16,24]. Nevertheless, the MEV in liver sinusoids decreased significantly 3 h after CLP and the MEV in postsinusoidal venules 10 h after CLP, which was a time delay to the observed reduction of PBF. Even at this time point, hepatic enzyme liberation increased significantly. One reason for this finding may be organ hypoxia caused by diminished liver perfusion. Nevertheless, it seems that hepatic microperfusion can be compensated by autoregulatory mechanisms or translocation of blood volume for a while. Constriction of hepatic stellate cells, which is mediated by endothelin-1, causes a decrease of sinusoidal diameter, which was observed 10 h after CLP in our study [15]. Therefore, changes of sinusoidal diameter influence the hepatic perfusion in already progressed stages of endotoxemia.

### Leukocyte-endothelial and platelet-endothelial interactions

Increased rolling of platelets in postsinusoidal venules was detected 1 h after CLP. Endothelial and platelet P-selectin, which can be rapidly released from storage granules, may be responsible for these findings [26]. Activated platelets and endotoxin stimulate the release of selectins from Weibel-Palade bodies, which induces rolling of leukocytes on endothelial cells [7,8,11,27,28]. Elevated leukocyte rolling in postsinusoidal venules was found 3 h after CLP. The involvement of platelets on leukocyte rolling recruitment explains the time lag between an increase of platelet and leukocyte rolling. Even in liver sinusoids, elevated amounts of sticking platelets could be detected 1 h after CLP, while significantly increased sticking leukocytes were found 5 h after CLP. A similar expectation was made in postsinusoidal venules where, 1 h after CLP, an increase of platelet sticking occurred. Nevertheless, significant elevated values of stickers of both cell types, leukocytes and platelets, were detected 5 h after CLP. These findings underline a crucial role of platelets in the initiation of leukocyte-endothelial interaction. Recently, an enhanced neutrophil adherence to endothelial cells in the presence of platelets and fibrinogen was described [30]. Our results confirming the initiation of leukocyte adherence to the endothelium by platelets are compatible with these findings. But the role of fibrinogen during these processes needs further evaluation. Platelet-leukocyte interaction is the first event of observable microcirculatory disturbances during endotoxemia. Reduced hepatic microperfusion occurs after the onset of leukocyte-endothelial interaction, which is initiated by platelet adherence. The increase of hepatocellular enzyme liberation is the result of hypoxia caused by decreased organ perfusion and the liberation of cytotoxic mediators (for example, superoxide, arachidonic acid metabolites, proteases) released by adherent and transendothelial migrated leukocytes. The continuous recruitment of platelets and leukocytes causes leukopenia and thrombopenia in blood count, as detected 3 h after CLP in our animal model. Thrombotic, non-perfused sinusoids increased significantly 5 h after CLP. The formation of stable platelet-leukocyte aggregates, which play an important role in thrombogenesis, may be responsible for this observation [29]. The role of β-integrins, which are responsible for leukocyte sticking on platelet endothelial adhesion, needs further investigation [9].

## Conclusion

We have demonstrated that hepatic platelet-endothelial adherence occurs early after the induction of endotoxemia. Leukocyte-endothelial interaction starts with a time delay to platelet adherence, which makes the involvement of platelets in the initiation of leukocyte-endothelial interaction probable. A decrease of hepatic microperfusion could be observed earlier in liver sinusoids than in postsinusoidal venules, but in both cases later than a reduction of PBF. Microcirculatory disturbances result in hepatocellular damage as a result of organ hypoxia and cytotoxic cellular damage.

## Key messages

• 	The hepatic microperfusion damage during endotoxemia follows a time course of ongoing processes.

• 	Platelet-endothelial adherence during endotoxemia in the liver is an early event.

• 	Leukocyte-endothelial adherence occurs after the onset of platelet-endothelial adherence.

• 	Decrease of liver perfusion is the consequence of inflammatory platelet and leukocyte adhesion.

• 	Hepatocellular damage is a combination of early toxic and late microperfusion related hepatocyte injury.

## Abbreviations

AST, ALT = hepatocellular enzymes; CLP = cecal ligation and puncture; FITC = fluorescein isothiocyanate; IVM = intravital microscopy; MAP = mean arterial pressure; MEV = mean erythrocyte velocity; PBF = portal blood flow.

## Competing interests

The authors declare that no financial or non-financial (political, personal, religious, ideological, academic, intellectual, commercial or any other) competing interests exist either now or in the future.

## Authors' contributions

RSC had the idea for the study, designed the study and supervised the research work. EH carried out the experiments. YK and MMG participated in the supervision of the experiments. TH assisted in establishing the staining of platelets. CH and EK participated in the study design and coordination. The authors read and approved the final manuscript. RSC and EH contributed equally to this work.

## Figures and Tables

**Table 1:** Macrohemodynamic parameters

	Time after cecal ligation and puncture					
	0 h	1 h	3 h	5 h	10 h	20 h

HR (beats/min)	358 (12)	365 (14)	369 (14)	340 (19)	331 (11)	339 (15)
MAP (mm Hg)	100 (6)	100 (5)	86 (4)	92 (4)	82 (2)^a^	81 (1)^a^
PBF (ml/minute)	17 (6)	9 (1)^a^	10 (1)^a^	9 (1)^a^	7 (1)^a^	6 (1)^a^

Values are means with standard error of the mean in parentheses. ^a^*p *< 0.05 versus 0 h. HR, heart rate; MAP, mean arterial pressure; PBF, portal blood flow.

**Table 2:** Body weight and laboratory values in venous blood of rats

	Time after cecal ligation and puncture					
	0 h	1 h	3 h	5 h	10 h	20 h

Weight (g)	247 (5)	231 (9)	233 (9)	249 (7)	220 (7)	219 (7)
WBC (× 10^3^/μl)	3.8 (0.3)	3.6 (0.2)	2.5 (0.2)^a^	2.4 (0.2)^a^	1.8 (0.2)^a^	2.0 (0.3)^a^
Plt (× 10^3^/μl)	843 (39)	900 (29)	750 (25)^a^	829 (26)	669 (25)^a^	488 (44)^a^
Hkt (%)	51 (1)	51 (1)	46 (2)	48 (2)	48 (2)	47 (4)
AST (U/l)	61 (9)	65 (4)	65 (4)	72 (9)	145 (26)^a^	234 (33)^a^
ALT (U/l)	30 (3)	27 (1)	24 (1)	29 (1)	61 (6)^a^	115 (11)^a^
Albumin (g/l)	12 (0.4)	12 (0.4)	11 (0.2)^a^	12 (0.3)	11 (0.2)^a^	10 (0.3)^a^
Bili (mg/dl)	0.32 (0.03)	0.26 (0.02)	0.29 (0.02)	0.23 (0.02)	0.26 (0.03)	0.48 (0.08)^a^

Values are means with standard error of the mean in parentheses. ^a^*p *< 0.05 versus 0 h. AST, ALT: hepatocellular enzymes; Bili: bilirubin; Hkt: haematokrit; Plt: platelets; WBC, white blood cell count.

**Table 3:** Intravital microscopy measurements in postsinusoidal venules and liver sinusoids

Measurement	Time point during intravital microscopy					
	0 h	1 h	3 h	5 h	10 h	20 h

MEV (mm/s)						
Venules	0.81 (0.03)	0.75 (0.03)	0.75 (0.04)	0.83 (0.03)	0.49 (0.02)^a^	0.40 (0.02)^a^
Sinusoids	0.39 (0.02)	0.38 (0.03)	0.29 (0.01)^a^	0.28 (0.02)^a^	0.25 (0.02)^a^	0.25 (0.02)^a^
Roller (mm^2 ^ES)						
Leukocytes	48 (16)	150 (36)	221 (49)^a^	111 (21)	222 (54)^a^	269 (69)^a^
Platelets	6 (4)	36 (7)^a^	37 (9)^a^	47 (11)^a^	38 (6)^a^	43 (9)^a^
Sinusoid diameter (μm)	8.00 (0.40)	7.90 (0.30)	7.20 (0.30)	7.30 (0.50)	7.00 (0.30)^a^	6.50 (0.30)^a^
Thrombotic sinusoids (%)	0 (0)	0 (1)	1 (1)	5 (1)^a^	8 (1)^a^	11 (2)^a^

Values are means with standard error of the mean in parentheses. ^a^*p *< 0.05 versus 0 h. ES, endothelial surface; MEV, mean erythrocyte velocity.
